# Evaluation of Human-Induced Pluripotent Stem Cells Derived from a Patient with Schwartz–Jampel Syndrome Revealed Distinct Hyperexcitability in the Skeletal Muscles

**DOI:** 10.3390/biomedicines11030814

**Published:** 2023-03-07

**Authors:** Yuri Yamashita, Satoshi Nakada, Kyoko Nakamura, Hidetoshi Sakurai, Kinji Ohno, Tomohide Goto, Yo Mabuchi, Chihiro Akazawa, Nobutaka Hattori, Eri Arikawa-Hirasawa

**Affiliations:** 1 Aging Biology in Health and Disease, Juntendo University Graduate School of Medicine, Tokyo 113-8421, Japan; 2 Department of Neurology, Faculty of Medicine, Juntendo University, Tokyo 113-8421, Japan; 3 Japanese Center for Research on Women in Sport, Juntendo University Graduate School of Health and Sports Science, Chiba 270-1695, Japan; 4 Department of Physiology, Juntendo University Graduate School of Medicine, Tokyo 113-8421, Japan; 5 Center for iPS Cell Research and Application (CiRA), Kyoto University, Kyoto 606-8507, Japan; 6 Division of Neurogenetics, Center for Neurological Diseases and Cancer, Nagoya University Graduate School of Medicine, Nagoya 466-8550, Japan; 7 Department of Neurology, Kanagawa Children’s Medical Center, Yokohama 232-8555, Japan; 8 Intractable Disease Research Centre, Juntendo University School of Medicine, Tokyo 113-8421, Japan

**Keywords:** Schwartz–Jampel syndrome, skeletal muscle, myotonia, perlecan, human-induced pluripotent stem cell, calcium imaging

## Abstract

Schwartz–Jampel syndrome (SJS) is an autosomal recessive disorder caused by loss-of-function mutations in heparan sulfate proteoglycan 2 (*HSPG2*), which encodes the core basement membrane protein perlecan. Myotonia is a major criterion for the diagnosis of SJS; however, its evaluation is based solely on physical examination and can be challenging in neonates and young children. Furthermore, the pathomechanism underlying SJS-related myotonia is not fully understood, and effective treatments for SJS are limited. Here, we established a cellular model of SJS using patient-derived human-induced pluripotent stem cells. This model exhibited hyper-responsiveness to acetylcholine as a result of abnormalities in the perlecan molecule, which were confirmed via comparison of their calcium imaging with calcium imaging of satellite cells derived from *Hspg2*^−/−^-Tg mice, which exhibit myotonic symptoms similar to SJS symptoms. Therefore, our results confirm the utility of creating cellular models for investigating SJS and their application in evaluating myotonia in clinical cases, while also providing a useful tool for the future screening of SJS therapies.

## 1. Introduction

Schwartz–Jampel syndrome (SJS), also known as chondrodystrophic myotonia, was originally described as a congenital blepharophimosis associated with a unique and generalized myopathy in 1962 [1]. Although SJS was conventionally classified into types 1a, 1b, and 2 according to clinical severity [2], it was later clarified that type 1 is caused by mutations in heparan sulfate proteoglycan 2 (*HSPG2*) [3] and type 2 by mutations in the leukemia inhibitory factor receptor [4]; thus, types 1 and 2 have been regarded as distinct diseases. Dyssegmental dysplagia Silvermann–Handmaker type (DDSH), another disease caused by mutations in *HSPG2*, is characterized by a complete loss of perlecan function and lack of extracellular perlecan expression, whereas SJS is associated with a partial loss of perlecan function and some extracellular expression of perlecan [5]. The lack of perlecan expression in DDSH causes severe vertebral disc separation and thoracic dysplasia, leading to perinatal lethality. SJS can also be viewed as a mild form of DDSH, as osteochondral dysplasia is milder in SJS than in DDSH and generalized muscle tone, including facial abnormalities, such as blepharophimosis or pursed lips, is prominent. There is another clinical classification of dyssegmental dysplasia wherein dyssegmental dysplagia Rolland–Desbuquois type (DDRD) is considered a mild form of DDSH [6], and there is confusion between the clinical and molecular classifications of these diseases. Recently, a patient with DDRD was reported to have a mutation in *HSPG2* [7], and the presence of myotonia was suggested; however, joint contracture made the evaluation of muscle symptoms difficult. An objective method of evaluating myotonia is examining the presence of continuous and spontaneous muscle activity at a high frequency and low amplitude, without any waxing or waning, using needle electromyography (nEMG) [8,9]. However, the invasiveness of nEMG in young children and the heterogeneity in the severity of myotonia in them make it difficult to diagnose and treat muscular abnormalities in younger patients. Furthermore, muscle biopsy and nerve conduction studies have not revealed any specific changes in these patients [10,11]. Thus, the difficulty in assessing SJS-induced myotonia, especially in the neonatal period, may lead to delayed risk management for myotonia that becomes evident with growth; therefore, a minimally invasive assessment tool needs to be established. In addition, there is still controversy over whether myotonia in SJS patients is neurogenic, myogenic, or derived from abnormalities at the neuromuscular junction, which makes development of effective treatments challenging.

Since the establishment of human-induced pluripotent stem cells (hiPSCs) in 2007 [12], disease organ models have been constructed using hiPSCs derived from patients with genetic diseases to elucidate pathological mechanisms [13,14]. Compared to mouse models, which have been mainly used to analyze pathological conditions, in vitro hiPSC models are more likely to reflect the pathological conditions without the differences in species between humans and mice. 

Calcium imaging has been used to determine cellular dynamics in various cells and tissues, including cardiac myocytes [15,16], skeletal muscles [17,18], and neurons [19,20], because calcium ions play an important role in the cellular functions of various organs, as they act as one of the major secondary messengers in most systems. 

Here, we investigated skeletal muscle abnormalities in SJS using a recording system of cytosolic Ca^2+^ dynamics in patient-derived hiPSCs. In addition, Ca^2+^ dynamics in myotubes from *Hspg2*^−/−^-Tg mice was compared to discuss the relationship between the extracellular molecule perlecan and muscle abnormalities, excluding neurogenic elements.

## 2. Materials and Methods

### 2.1. Establishing MyoD-hiPSCs

The hiPSCs were established using peripheral blood mononuclear cell samples obtained from a patient with SJS and two healthy volunteers using the episomal vector system as previously described [21]. A patient with SJS was known to harbor a heterozygous missense mutation, p.Leu1088Pro, in domain III-2 and a heterozygous nonsense mutation, p.Gln3061Ter, in domain IV of *HSPG2*; these mutations were predicted to be pathogenic using in silico analyses with S-VAR (http://p4d-info.nig.ac.jp/s-var/(accessed on 15 October 2021)), which is a prediction tool, with PolyPhen2, SIFT, PROVEAN, and PANTHER, and using in vitro analysis [22,23,24,25,26]. Muscle stiffness and chondrodysplasia were observed, and a needle electromyogram revealed continuous repetitive discharge [26]. These cells were then used to generate MyoD-hiPSCs that were able to undergo myogenic differentiation in response to tetracycline-induced MyoD overexpression, as previously described [27]. This produced two sets of MyoD-hiPSCs from each group for clonal validation, SJS_#10 and SJS_#15 from the patient samples, and Con_#10 and Con_#16 from healthy volunteers, which were confirmed by the expressions of *Oct3*/4, *Sox2*, and *Nanog*, which are pluripotent cell markers, and the ability to differentiate to tri-germ. These MyoD-hiPSCs used in this study were obtained from Center for iPS Cell Research and Application (CiRA), Kyoto University. All evaluations were performed after obtaining approval from the ethics review committees of Juntendo University (M08-0449) and Kyoto University (#R0091, #G259).

### 2.2. Myotube Differentiation Using MyoD-hiPSCs

**Figure 1:**
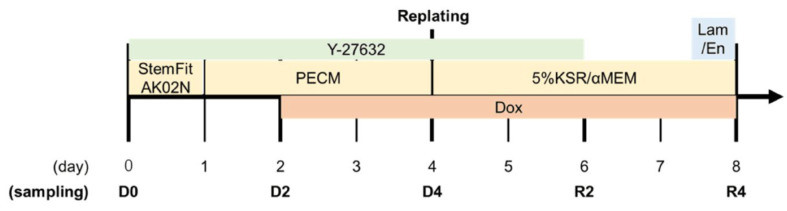
Scheme of the differentiation protocol of hiPSCs. PECM: primate ES cell medium, KSR: knockout serum replacement, Lam/En: laminin/entactin, Dox: doxycycline.

Culture and differentiation of hiPSCs were performed by modifying a previously described method [28]. Briefly, MyoD-hiPSCs were cultured on a feeder-free system using iMatrix-511 (Nippi, Tokyo, Japan)-coated plates and StemFit AK02N (Reprocell, Kanagawa, Japan) culture media. Myogenic differentiation of hiPSCs was induced by culturing cells in Primate ES Cell Medium (PECM) (Reprocell, Kanagawa, Japan) supplemented with 10 µM Y-27632 (FUJIFILM Wako Pure Chemical, Osaka, Japan), followed by culture in PECM supplemented with 0.3–1.6 µg/mL doxycycline (Dox; LKT laboratories, St. Paul, MN, USA) and 10 µM Y-27632. Differentiated hiPSCs were subsequently detached using Accutase (Nacalai Tesque, Kyoto, Japan) and replated on iMatrix-511-coated coverslips (5.7 × 10^4^ cells/cm^2^) and then differentiated into myotubes using 5% Knockout Serum Replacement (Thermo Fisher Scientific, Waltham, MA)/αMEM (Nacalai Tesque) supplemented with 200 µM 2-mercaptoethanol (2ME; Nacalai Tesque) and 0.3–1.6 µg/mL Dox. These differentiated myotubes derived from hiPSCs were evaluated 12–16 h after adding 70 µg/mL laminin/entactin complex (Corning Inc., Corning, NY, USA) and 10 ng/mL recombinant neural agrin (R&D Systems, Minneapolis, MN, USA). Scheme of the differentiation protocol of hiPSCs are shown in Figure 1.

For each cell line, the appropriate concentrations of Dox that induced differentiation without causing excessive cell death were determined via preliminary experiments and used in this study.

### 2.3. Experimental Animals

Satellite cells from skeletal muscle from perlecan conditional-knockout mice (*Hspg2*^−/−^-Tg mice) were used in this study to compare the findings between myotube derived from SJS-hiPSCs and myotube completely deficient of perlecan.

*Hspg2*^−/−^ mice, which lack perlecan expression due to disruption of the gene in exon 7 encoding the domain II region (N terminus of the LDL-receptor-like domain), exhibit perinatal lethality due to immature cartilage development, including cephalic hypoplasia [29]. Hence, a lethality-rescued mouse line (named *Hspg2*^−/−^-Tg with *Hspg2*^−/−^ and *Col2a1-Hspg2*^Tg/−^ genotypes) was established by crossing the transgenic mice with heterozygous *Hspg2*^+/−^ mice (named *Hspg2*^+/−^-Tg with *Hspg2*^+/−^ and *Col2a1*-*Hspg2*^Tg/−^ genotypes), which were created using a chondrocyte-specific *Col2a1* collagen promoter and enhancer [30]. Their genetic background was the same as that of *C57BL/6* mice. *Hspg2*^−/−^-Tg mice express perlecan only in the cartilage and exhibit blepharophimosis. An nEMG of *Hspg2*^−/−^-Tg mice revealed spontaneous and continuous firing of the muscle fibers similar to the competitive repetitive discharge observed in patients with SJS (Supplementary Figure S1). The mice were reared under a 12 h light–dark cycle at 23 ± 2 °C and fed a normal diet (CRF-1; Oriental Yeast Co., Ltd., Tokyo, Japan). All animal experiments were performed in accordance with the Fundamental Guidelines for Proper Conduct of Animal Experiments and Related Activities in Academic Research Institutions under the jurisdiction of the Ministry of Education, Culture, Sports, Science, and Technology (Notice No. 71, 2006) and were approved by the Committee for Animal Experimentation at Juntendo University (Approval No. 2022274).

### 2.4. Satellite Cell Culture and Differentiation

Quiescent satellite cells were collected from the skeletal muscles of 8-week-old male SJS-model mice using fluorescence-activated cell sorting and an SM/C-2.6 antibody, as described previously [31]. The SM/C-2.6 antibody was provided by Dr. Fukada. These satellite cells were seeded on rhLaminin-521 (Thermo Fisher Scientific)-coated plates and cultured in satellite cell growth media (Dulbecco’s modified Eagle’s medium (DMEM), 20% fetal bovine serum, 10% horse serum, chicken embryo extract (United States Biological, Salem, MA, USA), and penicillin/streptomycin) containing 2.5 µg/mL recombinant human FGF (ProteinTech, Rosemont, IL, USA), as previously described [32,33]. Satellite cells were differentiated to myotubes by culturing in high glucose DMEM (Thermo Fisher Scientific), supplemented with 2% horse serum (Thermo Fisher Scientific) and penicillin/streptomycin. To rescue perlecan-null myotubes, recombinant perlecan protein (20 μg/mL; purified and provided by Dr. Sasaki) was added at the start of the differentiation process. Calcium imaging experiments were performed 12–16 h after adding 70 µg/mL laminin/entactin complex (Corning Inc., Corning, NY, USA) and 10 ng/mL recombinant neural agrin (R&D Systems, Minneapolis, MN, USA).

### 2.5. Immunofluorescence Analysis

Cultured cells were washed in 1 × PBS for 10 min and fixed with 4% paraformaldehyde for 10 min at room temperature (RT). The cells were permeabilized with 0.2% triton-X for 10 min for myosin heavy chain (MHC) staining but not perlecan staining. After washing three times with 1 × PBS for 5 min, the cells were blocked with bovine serum albumin for 30 min at RT and incubated overnight with primary antibodies at appropriate dilutions. Next, they were washed three times with 1  ×  PBS for 10 min and then incubated with secondary antibodies. After washing with 1 × PBS for 10 min, the nuclei were stained with Hoechst 33258 (H3569; Thermo Fisher Scientific), and then, the cells were incubated with an autofluorescence inhibitor (Vector TrueVIEW Autofluorescence Quenching Kit, SP-8400; Vector Laboratories, Newark, CA, USA) for 5 min after washing with 1 × PBS for 5 min. Next, the cells were washed three times with 1 × PBS for 10 min each and then observed using a confocal fluorescence microscope. The images were analyzed using ImageJ software (2.3.0/1.53f, National Institutes of Health, Bethesda, MD, USA) [34]. The primary and secondary antibodies used are shown in Table 1.

### 2.6. Quantitative Real-Time PCR (qPCR)

The total RNA was extracted from cultured cells at 4 days after replating using Direct-zol RNA MiniPrep Plus (Zymo Research, Irvine, CA, USA). cDNA was synthesized from 500 ng RNA using ReverTra Ace qPCR RT Master Mix (TOYOBO, Osaka, Japan). qPCR was performed with Fast SYBR Green Master Mix (Thermo Fisher Scientific) on the Applied Biosystems (ABI) 7500 Fast-Real time PCR System (Applied Biosystems, Foster, CA, USA). Primer sets used in this study are described below (Table 2). Ribosomal protein S18 (*RPS18*) was used as the internal control.

### 2.7. Enzyme-Linked Immunosorbent Assay (ELISA)

To measure the expression levels of perlecan secreted from hiPSCs, ELISA was performed with cell culture supernatant collected on day 4 after replating using the Human HSPG2/Perlecan ELISA kit (Sandwich ELISA) (LS-F57116; LSBio**,** Seattle, WA, USA) according to the manufacturer’s protocol. The data were analyzed on a four-parameter logistic curve-fit using GainData (Arigo Biolaboratories, Hsinchu, Taiwan).

### 2.8. Calcium Imaging

Myotubes derived from both hiPSCs and satellite cells were washed with balanced salt solution (BSS) containing 135 mM NaCl, 5 mM KCl, 1 mM MgCl_2_, 2 mM CaCl_2_, 20 mM HEPES, and 10 mM glucose (pH 7.4). After washing, these myotubes were incubated with 5 μM fura-2-AM (Dojindo Laboratories, Kumamoto, Japan) in BSS at RT for 30 min.

Next, the fura-2-loaded iPSCs were washed twice in BSS, and calcium imaging was performed using an inverted fluorescence microscope equipped with a 20 × objective lens (S Fluor 20 × N.A. 0.75; Nikon, Tokyo, Japan) and AQUACOSMOS (Hamamatsu Photonics, Shizuoka, Japan) to acquire images, as previously described [35]. These fura-2 AM treated myotubes were excited with 340 nm and 380 nm excitation light, and the respective fluorescence intensities were acquired. The ratio of the two fluorescence intensities (340 nm/380 nm) was used as an index of intracellular Ca^2+^ influx. Acetylcholine (Ach) was perfused with BSS at a rate of 2 mL/min, and images were captured every 2 s. ACh was loaded at concentrations of 0.03, 0.1, 0.3, and 1.0 µM.

### 2.9. Statistical Analysis

The peaks induced by each ACh concentration were analyzed, and all values are presented as mean ± standard deviation (S.D.). Statistical differences were assessed using a two-way analysis of variance performed with GraphPad Prism 7 software (GraphPad Software, San Diego, CA, USA), and statistical significance was set at *p* < 0.05.

## 3. Results

### 3.1. Differentiation of Myotubes Derived from Control and SJS-hiPSCs

**Figure 2:**
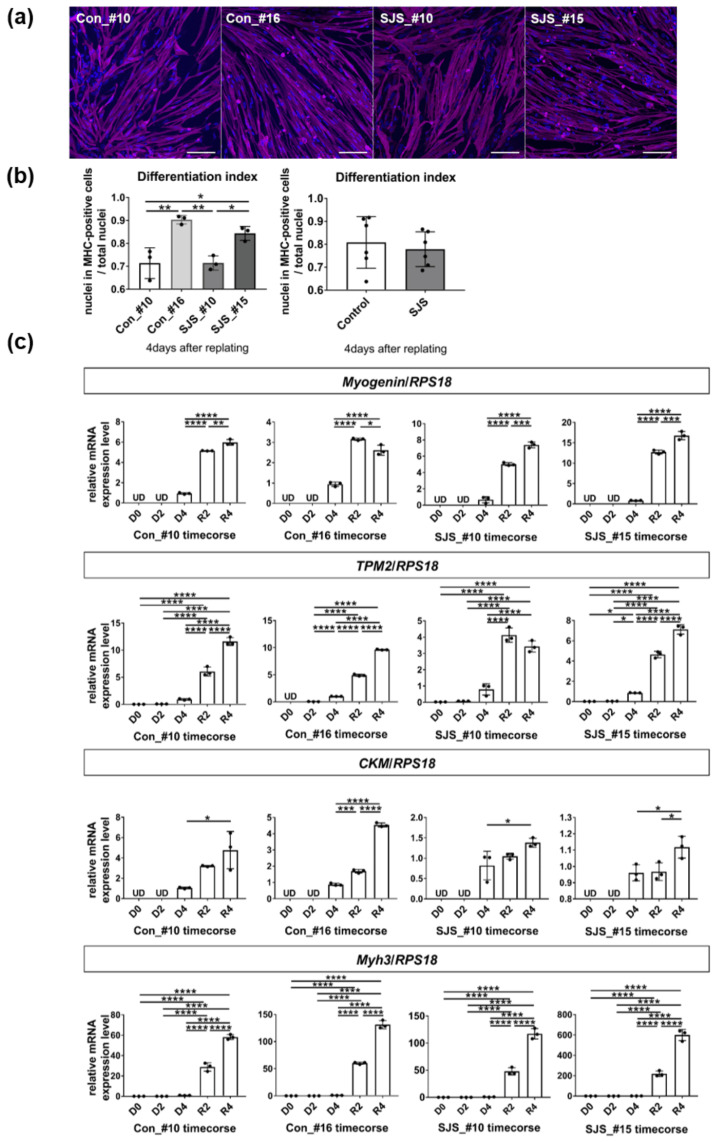
Differentiation of control and SJS-derived human-induced pluripotent stem cells (hiPSCs) into myotubular cells. (**a**) Myosin heavy chain (MHC) staining of myotubes derived from Con_#10, Con_#16, SJS_#10, and SJS_#15; pink shows MHC-positive cells and blue shows nuclei. (**b**) Differentiation index; the ratio of the number of nuclei in myotubes to the total number of nuclei. (**c**) The relative RNA expression levels of differentiation markers in skeletal muscle during differentiation. Mean and S.D. are indicated (*n*  =  3 in each clone). As shown in Figure 1, D0, D2, D4, R2, and R4 correspond to days 0, 2, 4, 6, and 8 after the initiation of differentiation, respectively. UD: undetectable. Data were analyzed using a two-tailed Student’s *t*-test (b) and one-way ANOVA with Tukey’s multiple comparison (b, c). *: *p*  <  0.05, **: *p* <0.01, ***: *p*  <  0.001, ****: *p*  <  0.0001. Scale bar, 100 μm.

The differentiation state of each clone was confirmed using the proportion of the number of nuclei in myotubes (MHC-positive cells) to the number of total cells, and the average proportion of SJS_#10 and SJS_#15 was not significantly different from the average proportion of Con_#16 and Con_#10, whereas the proportion of C16_#16 or SJS_#15 was higher than that of Con_10 or SJS_#10 (Figure 2a,b). In addition, changes in the expression levels of the differentiation markers in skeletal muscle, namely, myogenin, tropomyosin 2 (*TPM2*), creatine kinase-muscle (*CKM*), and myosin heavy chain 3 (*Myh3*), showed similar dynamics during differentiation among Con_#10, Con_#16, SJS_#10, and SJS_#15 (Figure 2c). Therefore, it was considered that all hiPSC lines could differentiate into myotubes; however, in order to consider the influence of the differentiation level of each strain, the following analyses were performed on all four strains.

### 3.2. Expression Level of Perlecan in Control- and SJS-hiPSCs

**Figure 3:**
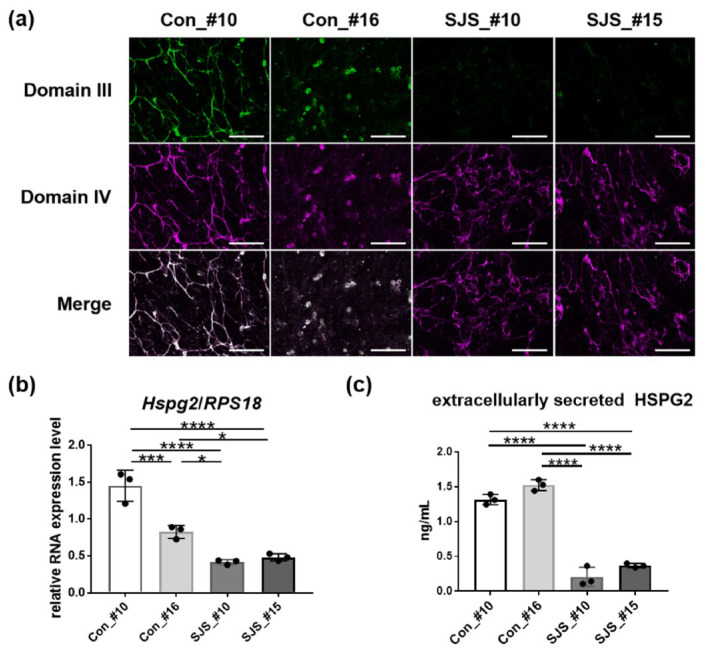
Perlecan expression in myotubes derived from control and SJS human-induced pluripotent stem cells (hiPSCs). (**a**) Immunofluorescence analysis of perlecan derived from Con_#10, Con_#16, SJS_#10, and SJS_#15. Green and pink indicate domains III and IV, respectively. (**b**) The relative RNA expression levels of *HSPG2* derived from hiPSCs. (**c**) The protein expression levels of perlecan extracellularly secreted from hiPSCs. Mean and S.D. are indicated (*n*  =  3 in each clone). Data were analyzed using a one-way ANOVA with Tukey’s multiple comparison (**b**,**c**). *: *p*  <  0.05, ***: *p*  <  0.001, ****: *p*  <  0.0001. Scale bar, 100 μm.

The immunofluorescence analysis of perlecan showed fibrous or mottled staining in myotubes derived from both control- and SJS-hiPSCs. Although the staining of domain IV was detected in both myotubes derived from control- and SJS-hiPSCs, domain III staining was weaker in SJS-hiPSCs than in control-hiPSCs (Figure 3a) (Supplementary Figure S2). qPCR performed with primer sets that recognize the beginning of *HSPG2* showed that the relative mRNA levels of *HSPG2* in SJS-hiPSCs (SJS_#10 and SJS_#15) were significantly lower than those in control-hiPSCs (Con_#10 and Con_#16) on day 8 of differentiation (Figure 3b). Furthermore, we also confirmed that the levels of perlecan protein extracellularly secreted from SJS-hiPSCs were also lower than those from control-hiPSCs by ELISA targeting domain V (Figure 3c). Thus, the mutations caused a decrease in both production and extracellular secretion, and these findings were consistent with those of a previous study [26].

### 3.3. Calcium Ion Dynamics in SJS-hiPSCs

**Figure 4:**
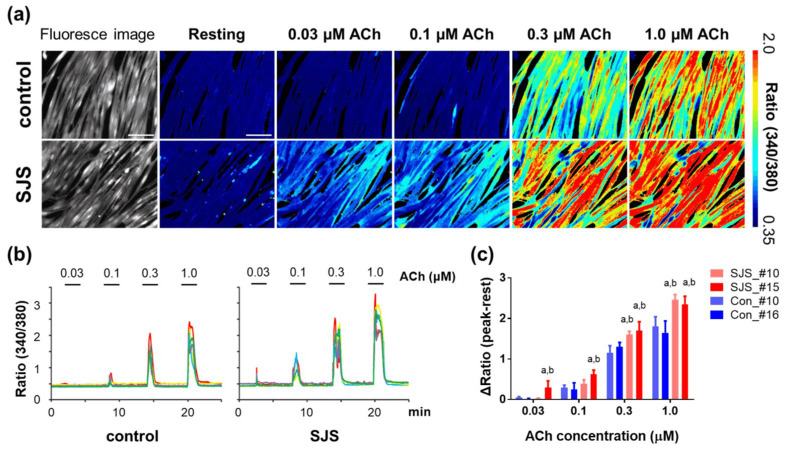
Ca^2+^ influx in Schwartz–Jampel syndrome (SJS) patient-derived and control human-induced pluripotent stem cell (hiPSC) myotubes. (**a**) Pseudocolor images of F340/F380 under both resting conditions and peak activity in response to increasing acetylcholine (ACh) concentration (0, 0.03, 0.1, 0.3, and 1.0 µM) in control and SJS-hiPSC myotubes. (**b**) Representative traces of Ca^2+^ influx induced by ACh (0, 0.03, 0.1, 0.3, and 1.0 µM) in control and SJS-hiPSC myotubes (*n* = 5). (**c**) Dose-response relationship between Ca^2+^ influx and ACh in control (Con_#10 and #16) and SJS hiPSC myotubes (SJS_#10 and #15). Data were evaluated using the two-way analysis of variance (ANOVA) and Tukey’s multiple comparison test (mean  ±  S.D., *n*  =  5–6). a. *p* < 0.05 vs. Con_#10, b. *p* < 0.05 vs. Con_#16. Scale bar, 100 µm.

We first investigated the probable skeletal muscle abnormalities in SJS using myotubes differentiated from hiPSCs. The increase in Ca^2+^ influx correlated with ACh dosage (0.03–1.0 µM) in both SJS and control hiPSC-derived myotubes, but this increase was higher in the SJS-hiPSCs than in the control-hiPSCs at each concentration of ACh (Figure 4). This trend only became significant at 0.3 and 1.0 µM ACh. These results suggest that the skeletal muscle in SJS can easily exhibit a transient increase in Ca^2+^ due to hyper-responsiveness to ACh, which might lead to the development of clinical symptoms, such as myotonia, in SJS. 

### 3.4. Calcium Ion Dynamics in Hspg2^−/−^-Tg Mouse Satellite Cell-Derived Myotubes

To date, around 30–40 mutations in *HSPG2* have been reported as pathogenic variants for SJS, with these mutations spread throughout the gene [26,36,37]. Based on this fact, we determined the calcium ion dynamics of perlecan-null myotubes derived from SJS model mouse satellite cells to avoid the specific effects of the *HSPG2* mutation in SJS patient-derived cells.

**Figure 5:**
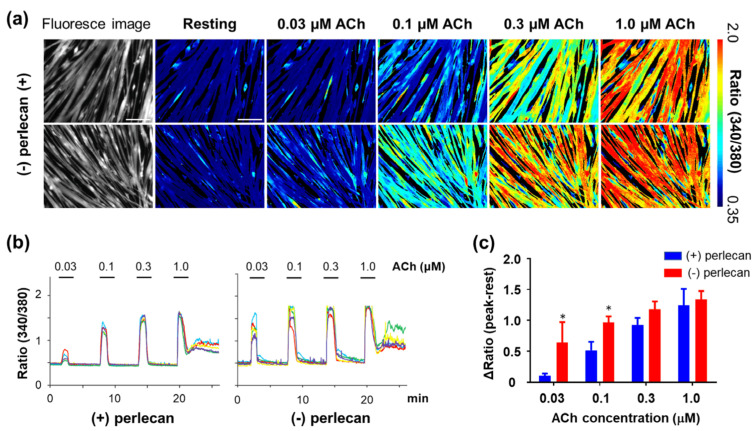
Comparison of Ca^2+^ influx in both perlecan-treated and untreated myotubes derived from *Hspg2*^−/−^-Tg mouse satellite cells. (**a**) Pseudocolor images of changes in F340/F380 at rest and peak stimulation in response to increasing concentrations of acetylcholine (ACh) (0, 0.03, 0.1, 0.3, and 1.0 µM) in both treated (with perlecan; upper panels) and untreated (without perlecan; lower panels) myotubes derived from SJS mice. (**b**) Representative traces of Ca^2+^ influx in response to ACh (0, 0.03, 0.1, 0.3, and 1.0 µM) in both treated (with perlecan) and untreated myotubes (*n* = 5). (**c**) Dose-response relationship between Ca^2+^ influx and ACh in both perlecan-treated and perlecan-null myotubes. Data were analyzed using the two-way analysis of variance (ANOVA) and Sidak’s multiple comparison test (mean  ±  S.D., *n*  =  3). * *p* < 0.05. Scale bar, 100 µm.

ACh induced a similar Ca^2+^ influx in these myotubes; however, the addition of recombinant perlecan significantly suppressed this response at lower concentrations of ACh, with these trends reaching significance at 0.03 and 0.1 µM ACh (*p* = 0.0081, at 0.03 µM; *p* = 0.0263, at 0.1 µM ACh) (Figure 5). We also confirmed that the addition of recombinant perlecan to WT myotubes did not change the Ca^2+^ influx level (Supplementary Figure S3). Thus, we infer that the ACh hyper-responsiveness observed in these myotubes is likely the result of the absence of functional perlecan.

## 4. Discussion

Perlecan is a major component of the extracellular matrix and is expressed in all basement membranes, including those of skeletal muscle cells. The five domains of perlecan bind to multiple proteins, including growth factors, such as fibroblast growth factor and platelet-derived growth factor, and cell surface receptors, such as alpha-dystroglycan and integrin beta 1 [38,39,40]. Therefore, perlecan plays an important role in the muscle abnormalities observed in patients with SJS, but the multifunctionality of perlecan makes it difficult to narrow down and identify the cause of persistent muscle activity in SJS, and definitive evaluation and treatment methods have not yet been developed.

Neurogenic and myogenic findings have been reported by electrophysiological studies in patients with SJS [41], and both presynaptic and synaptic abnormalities have been reported in vitro and in vivo using an animal model of SJS. For example, the abnormal myelination of presynaptic terminal nerves and the persistent axonal depolarization observed in SJS-model mice suggested some neurogenic factors in myotonia in SJS [42]. On the contrary, the importance of perlecan in neuromuscular junction (NMJ) formation associated with Wnt signaling [43], in the accumulation of acetylcholinesterase via collagen Q [44,45,46], and in promoting AChR clustering in the presence of laminin 2 [47] suggested that myotonia in SJS might be caused by the dysfunction of NMJ. In our previous study, lethality-rescued perlecan-knockout mice (*Hspg2*^−/−^-Tg mice) showed muscle irritability accompanied with increased insulin-like growth factor 1 and peroxisome proliferator-activated receptor-γ coactivator-1α expression and decreased myostatin expression in perlecan-deficient skeletal muscle [48,49]. However, studies on the validation of human clinical applications based on animal models remain limited.

Here, by evaluating calcium imaging data from SJS-hiPSC-derived myotubes, it was clarified that the skeletal muscle of patients with SJS exhibits a hypersensitive response to ACh, even in the absence of motor innervation. Myotonia is a symptom described as the prolonged contraction of skeletal muscles. The contraction of skeletal muscles is regulated by excitation–contraction coupling, which is a series of progressions from membrane depolarization triggered by ACh release from motor nerves to the interaction between actin and myosin via transient cytosolic Ca^2+^ [50,51,52]. In the present evaluation system, the neurogenic factor was excluded because the motor neurons were not included in this system. On the contrary, the reactivity of perlecan to ACh may be influenced by its ability to cluster AChE. In addition, defective perlecan that binds to dystroglycans may affect the integrity of sarcolemma or the function and localization of voltage-gated channels. It is expected that this evaluation system will help elucidate the pathogenesis of SJS and identify therapeutic targets in the future. Therefore, the hyperelevation of Ca^2+^ influx caused by ACh in SJS-derived myotubes might represent the major pathological mechanism underlying myotonia commonly observed in patients with SJS and may be useful for evaluating the presence of myotonia in clinical cases.

A limitation of the present study is that the degree of differentiation had to be artificially adjusted owing to differences in Dox reactivity of hiPSC clonal lines. Using an in vitro model, it has been reported that perlecan is required for myogenesis by binding to growth factors and that it is downregulated during differentiation [53]. Therefore, it is possible that mutations in *HSPG2* affect the proliferation and differentiation of SJS-hiPSCs; however, the degree of differentiation of the hiPSCs used in the present study did not differ sufficiently to outweigh the effects of differences in reactivity to Dox in each cell line. Furthermore, SJS-hiPSCs showed higher reactivity to ACh than control-hiPSCs, regardless of the differences in the degree of differentiation observed in MHC staining. Therefore, our results suggest high irritability of skeletal muscles in SJS. In addition, although only hiPSCs from one patient with SJS were evaluated, because of the rarity of this disease, similar hyper-responsiveness was observed in the perlecan-null myotubes of SJS-model mice, which exhibited blepharospasm and sustained muscle activity similar to in that in patients with SJS.

The SJS-hiPSCs used in this study had a heterozygous missense mutation, p.Leu1088Pro, in domain III-2 and a heterozygous nonsense mutation, *p*.Gln3061Ter, in domain IV of *HSPG2* and showed not only decreased expression of domains III and V, but also low mRNA expression of total *HSPG2.* This was a reasonable finding considering a previous report that the p.Leu1088Pro mutation decreases the production and secretion of perlecan [26]. In addition, the p.Leu1088Pro mutation in these SJS-hiPSCs might have altered the structure of domain III, preventing antibody recognition of this domain. Furthermore, the p.Gln3061Ter mutation in domain IV is a nonsense mutation causing loss of domain V, which binds to α-dystroglycan; thus, p. Gln3061Ter might inhibit the localization of perlecan to the plasma membrane and might also cause a decrease in *HSPG2* expression by nonsense-mediated mRNA decay. Thus, the hypersensitivity of skeletal muscle in patients with SJS is not caused by a complete absence of perlecan but possibly by the amount of perlecan. Our findings suggest that perlecan plays an important role in ensuring proper mechanical transduction without muscle collapse under excessive mechanical stimulation.

The average life expectancy of patients with SJS is generally unaffected by the condition. However, muscle abnormalities that worsen postural abnormalities in patients with chondrodysplasia may reduce the overall quality of life. Despite extensive research, no standard treatment is available for SJS-associated myotonia, and existing symptomatic treatments are also limited, with most treatments exhibiting only a partial effect. Therefore, it is critical to improve the tools used in the screening of effective drugs for skeletal muscle dysfunction in SJS. One such tool might be clinically relevant models, such as the one developed herein. In particular, our cellular model with Ca^2+^ imaging might help in developing effective treatments because many calcium indicators, including both chemical and genetically encoded indicators, have been developed and used for the evaluation of both pathophysiology and drug effectiveness using a real-time visual output [54,55]. In fact, excessive calcium influx has also been reported in other muscular dystrophy disorders such as DMD, and dystrophin expression by exon-skipping showed a suppressive effect on Ca influx in muscle cells derived from patients with DMD [56]. In addition, there are reports that suppressing ECM degradation related to Ca overload by using some drugs leads to an improvement in muscle fatigue in a *Caenorhabditis elegans* Duchenne muscular dystrophy model [57]; hence, our results will help to improve the detection of SJS pathogenesis and facilitate improved clinical intervention in the future.

## Figures and Tables

**Table 1:** Antibodies used for immunofluorescence analysis in this study.

Target	Primary Antibodies	Secondary Antibodies
MHC	Mouse anti-MHC monoclonal antibody (clone MF20, MAB4470; R&D Systems), dilution: 1/100	Alexa Fluor 647-labeled goat anti-mouse IgG (A21235; Thermo Fisher Scientific), dilution: 1/400
Perlecan	Rat anti-mouse Heparan Sulfate Proteoglycan (Perlecan) antibody (clone A7L6, MAB1948P; Merck), dilution: 1/200	Alexa Fluor 647-labeled goat anti-rat IgG (A11006; Thermo Fisher Scientific), dilution: 1/400
	Mouse anti-human perlecan monoclonal antibody (clone 7B5, 13-4400; Thermo Fisher Scientific), dilution: 1/200	Alexa Fluor 488-labeled goat anti-mouse IgG (A21235; Thermo Fisher Scientific), dilution: 1/400

MHC: myosin heavy chain.

**Table 2:** Sequence of primers used in this study.

Target	Sequence
* Myogenin*	Fw: TGGGCGTGTAAGGTGTGTAA
	Rev: CGATGTACTGGATGGCACTG
* TPM2*	Fw: ACGTGAGGACGAGCATGTG
	Rev: GTGCAGCGCTTGAGTGTCT
* CKM*	Fw: ACATGGCCAAGGTACTGACC
	Rev: TGATGGGGTCAAAGAGTTCC
* Myh3*	Fw: CTGGAGGATGAATGCTCAGAGC
	Rev: CCCAGAGAGTTCCTCAGTAAGG
* HSPG2*	Fw: GGCTGAGGGCATACGATGGCT
	Rev: CCCACTGCCCAGGTCGTCTCC
* RPS18*	Fw: GCAGAATCCACGCCAGTACAAG
	Rev: GCTTGTTGTCCAGACCATTGGC

*TPM2*: tropomyosin2, *CKM*: creatine kinase-muscle, *Myh3*: myosin heavy chain 3, *RPS18*: ribosomal protein S18.

## Data Availability

Not applicable.

## Supplementary Materials

The following supporting information can be downloaded at: https://www.mdpi.com/article/10.3390/biomedicines11030814/s1, Figure S1: Blepharophimosis and needle electromyogram findings in Hspg2−/−-Tg mice. Figure S2: Negative control of immunofluorescence analysis of perlecan in myotubes derived from control and SJS human-induced pluripotent stem cells (hiPSCs). Figure S3: Comparison of Ca2+ influx in both perlecan-treated and untreated myotubes derived from wild-type (Hspg2+/+) mouse satellite cells.
