# Supplementary material for: Evaluation of Human-Induced Pluripotent Stem Cells Derived from a Patient with Schwartz–Jampel Syndrome Revealed Distinct Hyperexcitability in the Skeletal Muscles
Source: Biomedicines. 2023 Mar 7;11(3):814. doi: 10.3390/biomedicines11030814 (PMC10045278; doi:10.3390/biomedicines11030814)
Supplement: Supplementary file 1 [file biomedicines-11-00814-s001.zip › biomedicines-2138863-supplementary.pdf]

Supplementary Figures

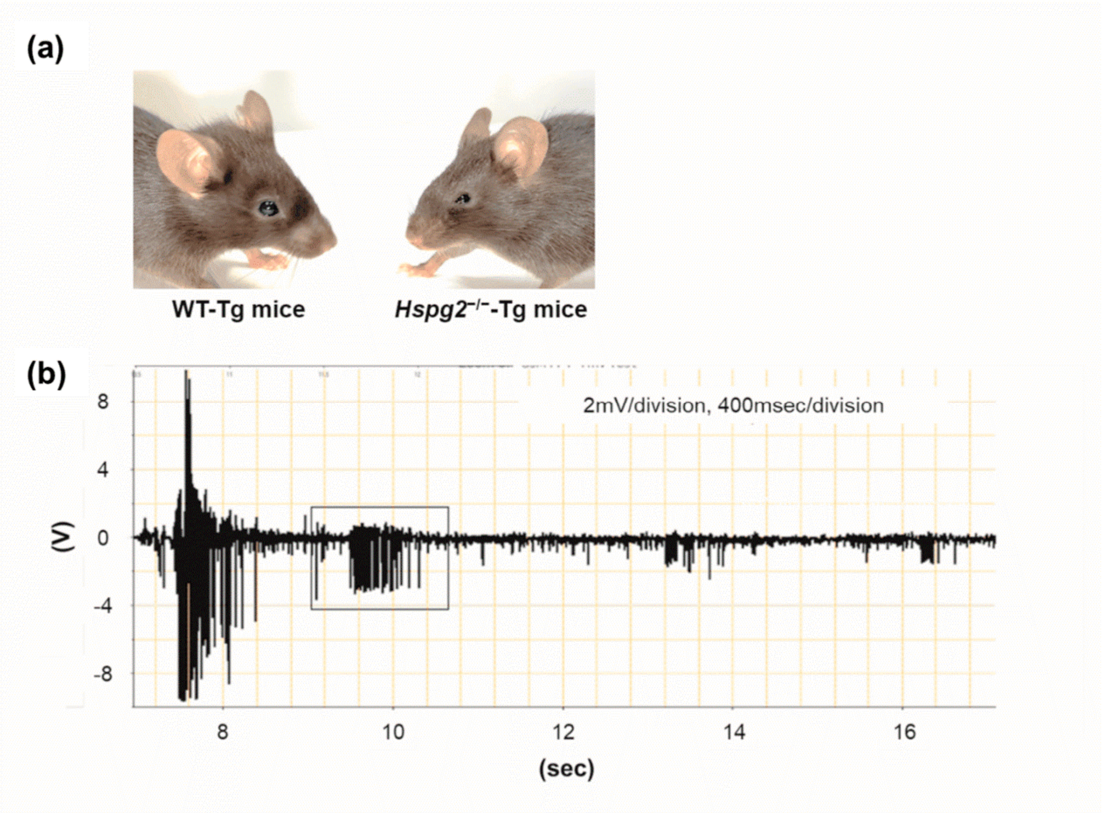

Supplementary Figure S1. (a) Blepharophimosis observed in *Hspg2*$^{-/-}$-Tg mice. ((right) *Hspg2*$^{-/-}$-Tg mice, (left) WT-Tg mice) **(b)** Spontaneous discharge revealed by needle electromyogram in *Hspg2*$^{-/-}$-Tg mice.

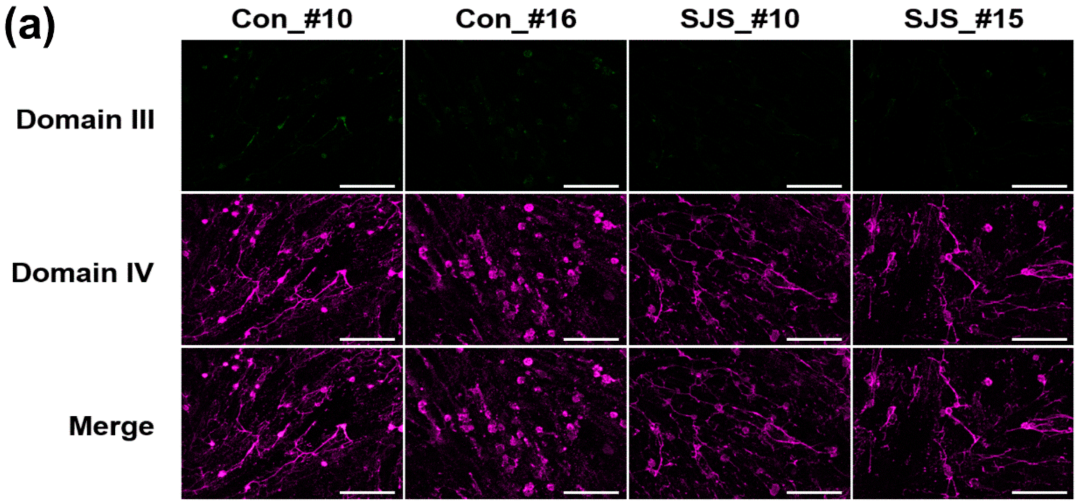

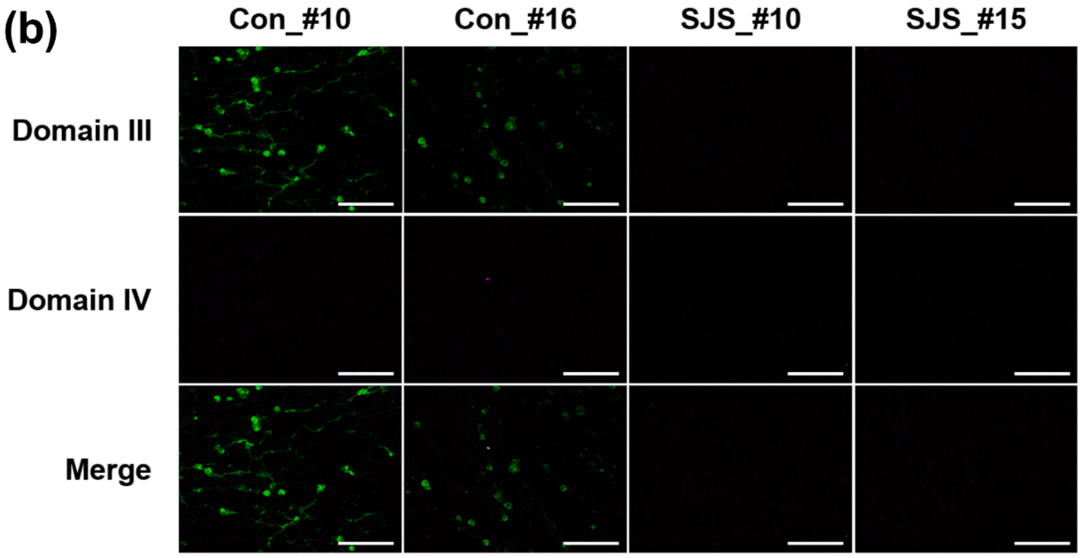

Supplementary Figure S2. Negative control of immunofluorescence analysis of perlecan in myotubes derived from control and SJS human-induced pluripotent stem cells (hiPSCs). Green and pink indicate domains III and IV, respectively. (a) Negative staining of the primary antibody against domain III in perlecan. (b) Negative staining of the primary antibody against domain IV in perlecan.

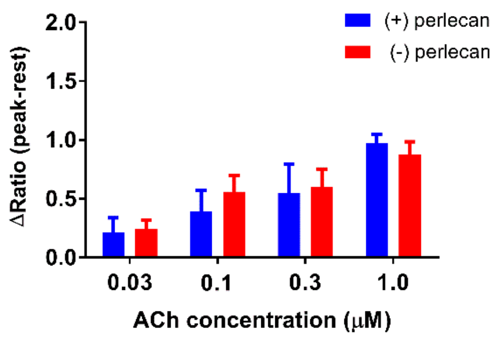

Supplementary Figure S3. Comparison of Ca^{2+} influx in both perlecan-treated and untreated myotubes derived from wild-type (*Hspg2*$^{+/+}$) mouse satellite cells. Cell culture, differentiation, and calcium imaging were performed similar to those of satellite cells derived from perlecan deficient satellite cells. Data were analyzed using the two-way analysis of variance (ANOVA) and Sidak's multiple comparison test (mean ± S.D., n = 3).
